# 2-Methoxystypandrone from *Polygonum cuspidatum* Rejuvenates Senescence by Reducing Mitochondrial ROS

**DOI:** 10.3390/antiox15030357

**Published:** 2026-03-11

**Authors:** Jee Hee Yoon, Ye Hyang Kim, Minseon Kim, Eun Young Jeong, Yun Haeng Lee, Ji Ho Park, Yoo Jin Lee, So Hun Lee, Ha Yeon Kim, Hye Min Kang, Hyung Wook Kwon, Youngjoo Byun, Song Seok Shin, Joon Tae Park

**Affiliations:** 1 Division of Life Sciences, College of Life Sciences and Bioengineering, Incheon National University, Incheon 22012, Republic of Korea; 2 Hyundai Bioland Co., Ltd., Cheongju-si 28162, Republic of Korea; dpgid27@hyundaibioland.co.kr (Y.H.K.); eyjeong99@hyundaibioland.co.kr (E.Y.J.); hayeonkim@hyundaibioland.co.kr (H.Y.K.);; 3 Convergence Research Center for Insect Vectors, Incheon National University, Incheon 22012, Republic of Korea; 4 College of Pharmacy, Korea University, Sejong 30019, Republic of Korea; yjbyun1@korea.ac.kr; 5 Interdisciplinary Major Program in Innovative Pharmaceutical Sciences, Korea University, Sejong 30019, Republic of Korea

**Keywords:** 2-methoxystypandrone, mitochondrial ROS, senescence amelioration, skin aging

## Abstract

Oxidative stress induced by reactive oxygen species (ROS) is a major contributor to senescence. Although strategies to mitigate ROS are considered crucial for reversing this process, effective interventions remain limited. Extracts from *Polygonum cuspidatum* (*P. cuspidatum*) have shown protective effects against senescence by suppressing mitochondrial ROS production; however, the specific bioactive compound responsible for these effects has not yet been identified. This study aimed to identify the active compound in *P. cuspidatum* responsible for reducing mitochondrial ROS and to elucidate its mechanism of action in rejuvenating senescence. Bioactive components of *P. cuspidatum* extract were screened for their ability to decrease mitochondrial ROS production. The most potent compound, 2-methoxystypandrone (2-MS), was further examined for its effects on oxidative phosphorylation (OXPHOS) efficiency, mitochondrial ROS generation, and senescence-associated phenotypes in a skin cell-based model. 2-MS was identified as the most effective compound for reducing mitochondrial ROS. Mechanistically, 2-MS enhanced OXPHOS efficiency, thereby minimizing ROS production resulting from inefficient respiration. Reduction in mitochondrial ROS by 2-MS restored senescence-associated phenotypes and rejuvenated senescence by suppressing ROS-driven melanogenesis and inflammatory responses in skin cells. This study identifies 2-MS as a key active ingredient of *P. cuspidatum* that exerts anti-aging effects through the reduction in mitochondrial ROS generation. These findings highlight 2-MS as a promising therapeutic and cosmetic candidate for rejuvenating senescence.

## 1. Introduction

Cellular senescence is a condition in which the cell cycle is permanently arrested due to diverse stressors, including oxidative stress-mediated damage, telomere shortening, and DNA damage [1]. Hallmarks of senescence include not only cell cycle arrest but also the progressive deterioration of organelle function [2]. Among these organelles, mitochondria are more susceptible to functional alterations during senescence [3]. Such mitochondrial dysfunction leads to electron leakage from the electron transport chain (ETC) in mitochondria, thereby promoting the production of reactive oxygen species (ROS). Physiological levels of ROS are essential for host defense, intracellular signaling pathways, and maintaining cellular homeostasis [4]. However, when ROS levels exceed physiological thresholds, they cause damage to cellular organelles [4]. Damage to cellular organelles by ROS is known to be a major cause of senescence [5]. Therefore, developing therapies that modulate mitochondrial ROS levels is a crucial strategy in anti-senescence research. Recently, MitoQ and MitoTEMPO, targeted antioxidants based on the triphenylphosphonium cation (TPP^+^) that selectively accumulate within mitochondria, have been developed. MitoQ, a lipid-soluble substance, resides primarily in the mitochondrial membrane and protects oxidative damage by inhibiting lipid peroxidation [6]. MitoTEMPO, on the other hand, is a mitochondrial-targeted antioxidant that scavenges superoxide radicals within the mitochondrial matrix [6]. However, these TPP^+^-based compounds have fundamental limitations stemming from their unique mechanism of action, known as membrane potential-dependent accumulation [7]. Excessive accumulation of MitoQ within mitochondria can severely overload the membrane potential, impairing membrane function [7]. Furthermore, MitoTEMPO exhibits a nonlinear dose–response relationship, potentially causing cytotoxicity even at doses slightly exceeding the optimal dose [8]. Consequently, existing TPP^+^-based ROS suppression strategies pose potential safety concerns. Therefore, to overcome these limitations, further research is essential for novel mitochondrial-targeted antioxidants that do not rely on the membrane potential accumulation mechanism of TPP^+^.

The skin aging process is complex and influenced by both internal and external factors [9]. Among the major biological processes contributing to skin aging are melanogenesis and inflammation [10,11]. Melanogenesis, the production of melanin in melanocytes, is primarily activated by ROS-mediated damage. ROS activates transcription factors including microphthalmia-associated transcription factor via mitogen-activated protein kinase pathways, thereby upregulating melanogenic enzymes including tyrosinase [12]. The process of melanin synthesis itself generates additional ROS, which exacerbate oxidative stress, damage cellular structures, and promote senescence of keratinocytes and fibroblasts [13]. In parallel, chronic low-grade inflammation in the skin, often referred to as ‘inflammaging’, represents another key driver of skin aging [10]. Excessive ROS activate redox-sensitive transcription factors, leading to the sustained expression of pro-inflammatory cytokines [14]. This persistent inflammatory signaling disrupts extracellular matrix integrity, resulting in wrinkle formation, reduced elasticity, and overall skin deterioration [14].

*Polygonum cuspidatum* (*P. cuspidatum*) is a medicinal plant known for potent anti-inflammatory and antioxidant effects [15]. It also provides significant dermatological benefits by regulating key enzymes associated with skin health [16]. Representative components of *P. cuspidatum* include polydatin, emodin-1-O-β-glucoside, emodin-8-glucoside, and 2-methoxystypandrone (2-MS) [15]. Recent studies have shown that polydatin plays a key role in the antioxidant effects of *P. cuspidatum* extracts [17]. However, little research has been conducted on the antioxidant properties of other components. Emodin-1-O-β-glucoside is a naturally occurring anthraquinone glycoside and a derivative of emodin. Emodin-1-O-β-glucoside is a potent non-competitive inhibitor of bacterial neuraminidase [18]. Emodin-8-glucoside exhibits antiviral effects and acts as an activator of peroxisome proliferator-activated receptor α/γ [19]. 2-MS is a natural naphthoquinone derivative extracted from the roots of *P. cuspidatum* [20,21]. 2-MS is known to possess various pharmacological activities, including anti-inflammatory, anticancer, antibacterial, and antiviral activities [22]. The discovery of an active ingredient with higher antioxidant activity than polydatin will further enhance the efficacy of existing polydatin-dependent treatments and expand therapeutic options as a combination therapy with polydatin.

In this study, we found that among the major components isolated from *P. cuspidatum* extract, 2-MS was the component that most significantly reduced mitochondrial ROS production. This reduction led to the recovery of senescence-associated phenotypes. Here, we propose that 2-MS is a promising new therapeutic candidate for rejuvenating senescence.

## 2. Materials and Methods

### 2.1. Cell Culture

Human dermal fibroblasts (PCS-201-010; ATCC, Manassas, VA, USA) were used. To induce cell senescence, cells were serially passaged following the previously described protocols [23]. The following criteria were applied to distinguish senescent fibroblasts from young fibroblasts. Cells with a population doubling time of 14 days or more were classified as senescent fibroblasts, and cells with a population doubling time of less than 2 days were classified as fibroblasts [23,24,25,26]. Moreover, cells with a senescence-associated β-Galactosidase (SA-β-Gal) positivity of 70% or more were classified as senescent fibroblasts, and cells with a positivity of less than 3% were classified as young senescent fibroblasts [26] (Figure S1A). Finally, senescent and young fibroblasts were classified based on the finding that the expression of antiproliferative markers *p21* and *p16* was significantly lower in young fibroblasts than in senescent fibroblasts [27] (Figure S1B,C). B16-F1 cells (CRL-6323; ATCC), immortalized human keratinocytes (HaCaT; 300493; Cytion, Eppelheim, Germany), and Raw264.7 macrophages (40071; Korea Cell Line Bank, Seoul, Republic of Korea) were used.

Dulbecco’s modified Eagle’s medium (MD10000A; SPL Life Sciences, Pocheon, South Korea; DMEM) was used to culture all cells. DMEM was supplemented with 25 mM glucose, 10% fetal bovine serum (35–015–CV; Corning, Corning, NY, USA), 100 μg/mL penicillin, and 100 μg/mL streptomycin (SV30079.01; Hyclone, Logan, UT, USA) [17].

### 2.2. Flow Cytometric Analysis of Mitochondrial ROS

Mitochondrial ROS levels were measured in senescent fibroblasts treated with DMSO (D8418; Sigma, St. Louis, MO, USA, 0.01%), emodin-1-O-β-glucoside (CFN93238; ChemFaces, Wuhan, China, 8 μM), emodin-8-glucoside (HY-N0311; MedChemExpress, Monmouth, NJ, USA, 8 μM), 2-MS (CFN97412; ChemFaces, 8 μM), or polydatin (15721-25G; Sigma, 8 μM) for 12 days at 4-day intervals. After that, cells (5 × 10^3^) were treated for 30 min at 37 °C in media containing 30 µM dihydrorhodamine 123 (10056-1; Bi-otium, Fremont, CA, USA, DHR123). Negative control cells were incubated under identical conditions in DHR123-free media. DHR123 is oxidized by ROS to form the fluorescent dye rhodamine 123, which accumulates in mitochondria in a membrane potential–dependent manner, allowing the assessment of mitochondrial ROS levels [28]. Mitochondrial ROS levels were measured on an automated flow cytometry system (CytoFLEX; Beckman Coulter Life Sciences, Indianapolis, IN, USA). Flow cytometry data were analyzed using CytExpert software (version 2.4; Beckman Coulter Life Sciences). The mean fluorescence intensity is expressed as MFI. Mitochondrial ROS was calculated using the following formula: [FITC MFI after DHR123 staining] − [FITC MFI after unstained DHR123].

### 2.3. Neutral Comet Assay

Senescent fibroblasts were treated with 2-MS (4, 8, or 12 μM) or DMSO (0.01%) at 4-day intervals for 12 days. DNA tail length was measured using the CometAssay Single Cell Gel Electrophoresis Assay Kit (4250–050–K; R&D systems, Minneapolis, MN, USA). The manufacturer’s instructions were followed.

### 2.4. Oxygen Consumption Rate (OCR), Proton Leak, Extracellular Acidification Rate (ECAR), and Basal Proton Efflux Rate

Senescent fibroblasts or young fibroblasts were treated with DMSO (0.01%) or 2-MS (4, 8, and 12 μM) at 4-day intervals for 12 days to assess oxygen consumption rate (OCR; pmol/min), proton leak, extracellular acidification rate (ECAR), and basal proton efflux rate. OCR and proton leak were measured using the Seahorse XF Mito Stress Test Kit (103015-100; Agilent Technologies, Santa Clara, CA, USA). ECAR and basal proton efflux rate were measured using the Seahorse XF Glycolytic Rate Assay Kit (103344-100; Agilent Technologies). All procedures were performed in compliance with the manufacturer’s instructions and previously discussed techniques [29].

### 2.5. Flow Cytometric Analysis of Mitochondrial Membrane Potential (MMP) and Mitochondrial Mass

MMP and mitochondrial mass were measured in senescent or young fibroblasts treated with DMSO (D8418; Sigma, 0.01%) and 2-MS (CFN97412; ChemFaces, 4, 8, and 12 μM) at 4-day intervals for 12 days. For MMP and mitochondrial mass measurements, cells were exposed to medium containing 0.6 μg/mL JC-10 (ENZ-52305; Enzo Life Sciences, Farmingdale, NY, USA) and 50 nM MitoTracker™ Deep Red FM dye (MTDR; M46753; Invitrogen, Waltham, MA, USA) for 30 min at 37 °C. MMP and mitochondrial mass were measured and calculated according to the manufacturer’s instructions.

### 2.6. Immunofluorescence Analysis

As previously mentioned, immunofluorescence analysis was carried out [29]. Briefly, primary antibodies comprised mouse anti-OXPHOS cocktail (ab110411; Abcam, Cambridge, UK; 1:200 dilution) and rabbit anti-LC3B (A19665; Abclonal, Boston, MA, USA; 1:200 dilution). Alexa Fluor^®^ 647–conjugated goat anti–mouse IgG (A–28181; 1:200; Invitrogen) and Alexa Fluor^®^ 488–conjugated goat anti–rabbit IgG (A–11008; 1:200; Invitrogen, Carlsbad, CA, USA) were used as secondary antibodies.

### 2.7. Flow Cytometric Analysis of Autophagy Flux and Autofluorescence

Senescent fibroblasts were treated for 12 days at 4-day intervals with either 2-MS (4, 8, and 12 μM) or DMSO (0.01%). To evaluate autophagy flux, 20 μM chloroquine (CQ; C6628; Sigma, St. Louis, MO, USA) was applied (w/) or not (w/o) 24 h before flow cytometry. Then, Cyto-ID staining solution (ENZ-51031-0050; Enzo Life Sciences) was used following the manufacturer’s direction. To evaluate autofluorescence, dye-free media were applied for 30 min at 37 °C. Subsequent experimental procedures followed those of previous studies [29].

### 2.8. Senescent-Associated β–Galactosidase (SA–β–Gal) Staining

Senescent fibroblasts were treated for 12 days at 4-day intervals with either 2-MS (4, 8, and 12 μM) or DMSO (0.01%). For SA-β-Gal staining (9860; Cell Signaling Technology, Beverly, MA, USA), the manufacturer’s instructions were adhered to.

### 2.9. Preparation of Complementary DNA (cDNA)

Senescent fibroblasts or young fibroblasts were treated for 12 days at 4-day intervals with either 2-MS (4, 8, and 12 μM) or DMSO (0.01%). Using the Rneasy Mini Kit (74104; QIAGEN, Hilden, Germany), total RNA was extracted from 1 × 10^6^ cells in accordance with the manufacturer’s instructions. The DiaStar^TM^ RT Kit (DR22-R10k; SolGent, Seoul, Korea) was used for reverse transcription to create complementary DNA (cDNA).

### 2.10. Quantitative PCR (qPCR) Analysis

qPCR was carried out following the procedure in our previous study [29]. qPCR was done using primers in Table 1.

### 2.11. Western Blot Analysis

Western blot analysis was carried out in accordance with Table 2’s instructions.

### 2.12. ROS Assay Using HaCaT Keratinocytes

HaCaT keratinocytes were treated with DMSO (0.01%), 2-MS (0.1, 0.5, and 1 μg/mL; 0.384, 1.92, and 3.84 μM), or EGCG (1.5 μM) for 1 day. Then, HaCaT keratinocytes were exposed with hydrogen peroxide (516813; Sigma, H_2_O_2_) for 30 min. ROS levels were measured using 20 μM 2′,7′-dichlorodihydrofluorescein diacetate (D6883; Sigma, USA). Fluorescence was measured at 485/535 nm using a microplate reader Spark 10M (Tecan, Männedorf, Switzerland).

### 2.13. Melanin Production and Secretion Assay

1 × 10^6^ B16-F1 cells were stimulated with 100 nM α-melanocyte-stimulating hormone (M4135; Sigma, α-MSH) for 3 days. Then, B16-F1 cells were treated with DMSO (0.01%), 2-MS (0.1, 0.5, and 1 μg/mL; 0.384, 1.92, and 3.84 μM), or arbutin (50 μg/mL) for 1 day. To measure the rate of melanin production, B16-F1 cells were collected and treated with 120 μL of 1N NaOH (1636; Duksan, Ansan, Republic of Korea). The cell lysate was then incubated at 60 °C for 30 min. The rate of melanin production was measured at 450 nm using a microplate reader Spark 10M (Tecan). To measure the rate of melanin release, supernatants were collected. The rate of melanin production was measured at 450 nm using a microplate reader Spark 10M (Tecan).

### 2.14. Inducible Nitric Oxide Synthase (iNOS) Assay

RAW264.7 macrophages were stimulated with 0.1 μg/mL lipopolysaccharide (L4516; Sigma LPS). Then, RAW264.7 macrophages were treated with DMSO (0.01%), 2-MS (3.84, 19.2, 38.4, and 96 μM), or indomethacin (75 μg/mL) for 1 day. The iNOS activity was measured using Griess assay kit (MAK36, Sigma).

### 2.15. Statistical Analysis

All statistical analyses were conducted using GraphPad Prism 9 (GraphPad Software, San Diego, CA, USA). Differences between groups were evaluated using one-way ANOVA or two-way ANOVA, followed by Bonferroni’s post hoc test.

## 3. Results

### 3.1. 2-Methoxystypandrone Inhibits Mitochondrial ROS Production in Senescent Fibroblasts

**Figure 1:**
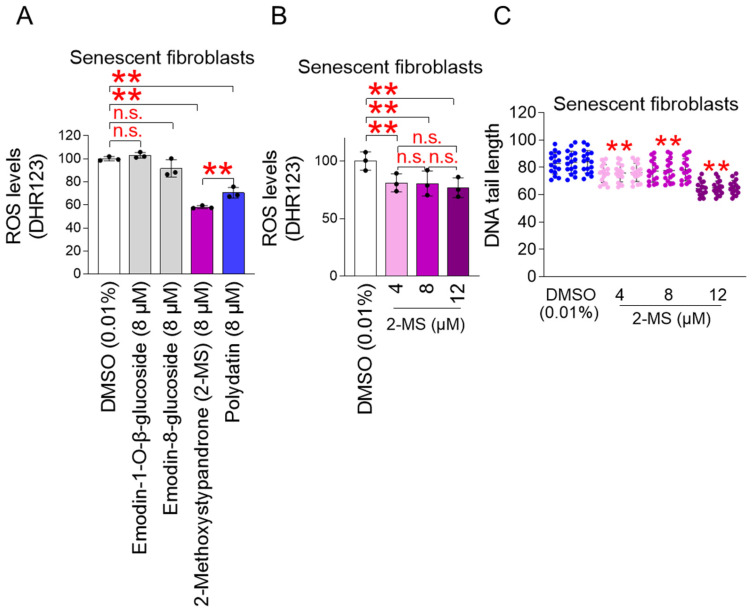
2-methoxystypandrone (2-MS) inhibits mitochondrial ROS production in senescent fibroblasts. (**A**) Senescent fibroblasts treated with DMSO (0.01%), emodin-1-O-β-glucoside (8 μM), emodin-8-glucoside (8 μM), 2-methoxystypandrone (2-MS) (8 μM), or polydatin (8 μM) for 12 days at 4-day intervals. Mitochondrial ROS were measured after staining cells with 30 µM dihydrorhodamine 123 (DHR123. Statistical analysis was performed using one-way ANOVA followed by Bonferroni’s post–hoc test, with results considered not significant (n.s.) or significant at ** *p* < 0.01. Data represent the mean ± S.D., *n* = 3. (**B**) Senescent fibroblasts treated with DMSO (0.01%) or 2-MS (4, 8, and 12 μM) for 12 days at 4-day intervals. Mitochondrial ROS were measured after staining cells with 30 µM DHR123. Statistical analysis was performed using one-way ANOVA followed by Bonferroni’s post–hoc test, with results considered not significant (n.s.) or significant at ** *p* < 0.01. Data represent the mean ± S.D., *n* = 3. (**C**) Senescent fibroblasts treated with DMSO (0.01%) or 2-MS (4, 8, and 12 μM) for 12 days at 4-day intervals. DNA tail length was measured using the neutral comet assay. Statistical analysis was performed using one-way ANOVA followed by Bonferroni’s post–hoc test, with results considered significant at ** *p* < 0.01. For each condition, the experiment was repeated three times, yielding three independent sets of data (biological triplicates). Sixteen different samples were analyzed to generate each biological triplicate. Data represent the mean ± S.D.

A screening procedure was performed to select the active ingredient of *P. cuspidatum* extract with greater antioxidant activity than polydatin (the active ingredient of the extract) [17]. Emodin-1-O-β-glucoside, emodin-8-glucoside, and 2-methoxystypandrone (2-MS), known as the main components of *P. cuspidatum* extract, were selected as test subjects [15]. Polydatin was used as a positive control. Because polydatin exhibits proven antioxidant effects at 8 μM [17], test compounds were assessed at the same concentration. Senescent fibroblasts were treated with 8 μM of each compound every 4 days for a total of 12 days, followed by the measurement of mitochondrial ROS levels. As expected, compared with the DMSO control, polydatin dramatically reduced mitochondrial ROS levels in senescent fibroblasts (Figure 1A). Compared with the DMSO control, emodin-1-O-β-glucoside and emodin-8-glucoside failed to reduce mitochondrial ROS levels in senescent fibroblasts (Figure 1A). However, compared with the DMSO control, 2-MS significantly reduced mitochondrial ROS levels (Figure 1A). Furthermore, compared with polydatin, 2-MS significantly reduced mitochondrial ROS levels (Figure 1A). These results suggest that 2-MS, among the active components of *P. cuspidatum* extracts, possesses stronger antioxidant activity than polydatin.

To rule out the possibility that 2-MS was effective in reducing mitochondrial ROS only at the 8 μM concentration used in the screening, we examined whether 2-MS also reduced mitochondrial ROS at concentrations other than 8 μM, including 4 μM and 12 μM. At all concentrations used (4, 8, and 12 μM), 2-MS significantly reduced mitochondrial ROS levels compared to the DMSO control (Figure 1B). There was no significant difference in the degree of mitochondrial ROS reduction in senescent fibroblasts at concentrations of 4, 8, and 12 μM (Figure 1B). To thoroughly examine the anti-senescence potential of 2-MS, subsequent experiments were carried out across all three concentrations.

ROS can damage DNA directly or damage proteins involved in DNA maintenance [30]. Our finding that 2-MS reduces mitochondrial ROS production led us to investigate the effects of 2-MS on DNA damage. DNA damage was assessed by measuring DNA tail length, which reflects the extent of damage. At all concentrations used (4, 8, and 12 μM), 2-MS significantly reduced DNA DSBs compared to the DMSO control (Figure 1C). These results confirmed the effect of 2-MS in reducing mitochondrial ROS production.

### 3.2. 2-MS Restores Mitochondrial Function

**Figure 2:**
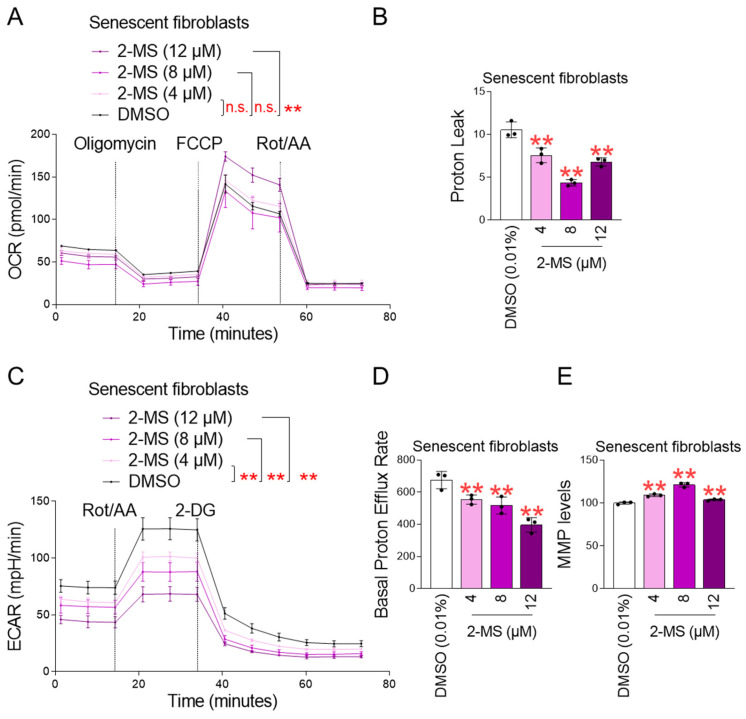
2-MS restores mitochondrial function in senescent fibroblasts. Senescent fibroblasts treated with DMSO (0.01%) or 2-MS (4, 8, and 12 μM) for 12 days at 4-day intervals. Oxygen consumption rate (OCR; pmol/min) and proton leak were measured using the Seahorse XF Mito Stress Test Kit (**A**,**B**). ECAR and basal proton efflux rate were measured using the Seahorse XF Glycolytic Rate Assay Kit (**C**,**D**). Flow cytometric analysis of mitochondrial membrane potential (MMP) using JC-1 (**E**). Statistical significance of OCR and ECAR was assessed using two-way ANOVA followed by Bonferroni’s post hoc test, with results considered not significant (n.s.) or significant at ** *p* < 0.01. Data represent the mean ± S.D., *n* = 5. Statistical significance of proton leak, basal proton efflux, and MMP levels were assessed using one-way ANOVA followed by Bonferroni’s post hoc test, with results considered significant at ** *p* < 0.01. Data represent the mean ± S.D., *n* = 3.

One of the main causes of ROS production is inefficiency in electron transport in the mitochondrial electron transport chain (ETC) [31]. In particular, impaired electron flow within ETC complexes promotes the reduction of oxygen to superoxide radicals [31]. The efficiency of oxidative phosphorylation (OXPHOS) is commonly used as a measure of electron transport performance [32]. To investigate the mechanism by which 2-MS reduces ROS, we assessed OXPHOS efficiency by measuring the oxygen consumption rate (OCR) [33]. Sequential injections of oligomycin, carbonyl cyanide-p-trifluoromethoxyphenylhydrazone (FCCP), and rotenone/antimycin A assessed non-mitochondrial respiration, maximal respiration, and ATP-coupled respiration, respectively [34]. Compared to DMSO-treated senescent fibroblasts, 2-MS treatment (4, 8, and 12 μM) did not alter non-mitochondrial respiration (Figure S2A). Furthermore, 4 and 8 μM 2-MS did not alter maximal respiration compared to DMSO-treated senescent fibroblasts, whereas 12 μM 2-MS significantly increased maximal respiration compared to the control (Figure S2B). Furthermore, 4 μM 2-MS did not alter ATP-coupled respiration compared to the control, whereas 8 μM and 12 μM 2-MS significantly reduced ATP-coupled respiration compared to the control (Figure S2C). Because it was difficult to obtain clues about the effect of 2-MS on electron transport efficiency through analysis of individual respiratory parameters (non-mitochondrial respiration, maximal respiration, and ATP-coupled respiration), we focused on the effect of 2-MS on the overall efficiency of OXPHOS. Treatment with 12 μM 2-MS significantly increased OCR compared to DMSO-treated senescent fibroblasts, indicating enhanced OXPHOS efficiency (Figure 2A). In contrast, 4 μM and 8 μM 2-MS did not significantly alter OCR relative to controls (Figure 2A). These findings suggest that 12 μM 2-MS is sufficient to improve OXPHOS efficiency, whereas lower concentrations are ineffective.

The finding that 2-MS treatment did not increase OXPHOS efficiency at low concentrations (4 μM and 8 μM) led us to investigate proton leak, which represents inefficient electron transport. 2-MS treatment at concentrations of 4, 8, and 12 μM significantly reduced proton leak in senescent fibroblasts compared to the DMSO control, suggesting efficient electron transport (Figure 2B).

Efficient OXPHOS reflects a reduced r reliance on glycolysis for an energy source [35]. To assess glycolytic activity, the extracellular acidification rate (ECAR) was measured before/after treatment with rotenone/antimycin A and 2-deoxy-D-glucose (2-DG) [33]. 2-MS treatment significantly reduced ECAR at all tested concentrations (4, 8, and 12 μM) compared with DMSO-treated senescent fibroblasts (Figure 2C). These findings suggest that 2-MS suppresses glycolytic activity, thereby decreasing dependence on glycolysis.

Lactic acid is produced during glycolysis through the use of excess protons not required for cellular respiration [36]. This process, known as lactic acid fermentation, is an inefficient metabolic pathway that generates only limited energy [36]. Furthermore, the conversion of pyruvate to lactic acid generates additional protons [37]. To evaluate the effect of 2-MS on this inefficient pathway, the basal proton efflux rate was measured. Compared with DMSO-treated senescent fibroblasts, 2-MS treatment (4, 8, and 12 μM) significantly reduced the basal proton efflux rate, indicating that 2-MS suppresses lactic acid fermentation (Figure 2D).

Next, we investigated the effect of 2-MS on mitochondrial membrane potential (MMP), which is affected by ROS-induced mitochondrial damage [38]. 2-MS treatment (4, 8, and 12 μM) significantly improved MMP compared to the DMSO control, indicating that the reduction in mitochondrial ROS production by 2-MS leads to the restoration of MMP (Figure 2E).

Having established that 2-MS restores mitochondrial function in senescent fibroblasts, we investigated whether 2-MS also modulates it in young fibroblasts. At high concentrations (8 μM and 12 μM), 2-MS exhibited OCR that was indistinguishable from that of DMSO-treated young fibroblasts, but at a low concentration (4 μM), OCR levels were significantly reduced (Figure S3A). Furthermore, proton leak remained unchanged at all 2-MS concentrations (4, 8, and 12 μM), indicating that 2-MS had a minimal effect on electron transport efficiency in young fibroblasts [39] (Figure S3B). Next, we investigated the effect of 2-MS on glycolytic metabolism. Treatment with 4 and 12 μM 2-MS significantly decreased ECAR, but at 8 μM, no significant effect was observed compared to DMSO-treated young fibroblasts (Figure S3C). Furthermore, the basal proton efflux rate was significantly reduced only at the highest concentration, 12 μM, indicating that 2-MS at this concentration was highly effective in inhibiting glycolytic metabolism (Figure S3D). Finally, the effect of 2-MS on MMP was assessed. In young fibroblasts, 2-MS (4, 8, and 12 μM) did not alter MMP compared to DMSO-treated young fibroblasts (Figure S3E). This result may be due to the fact that young fibroblasts already have an optimal basal electrochemical gradient due to minimal mitochondrial damage caused by ROS [40].

### 3.3. 2-MS Eliminates Dysfunctional Mitochondria Through Mitophagy

The mechanism of 2-MS’s beneficial effects was further explored after observing its role in restoring mitochondrial function. Damaged mitochondria are removed through mitophagy, a process that contributes to mitochondrial quality control [41]. Thus, we hypothesized that 2-MS restores mitochondrial activity by promoting mitophagy. To verify this, we examined the colocalization of mitochondria with microtubule-associated protein 1A/1B-light chain 3B (LC3B), a membrane protein of autophagosomes, as mitophagy selectively removes dysfunctional mitochondria via autophagosomes [42].

**Figure 3:**
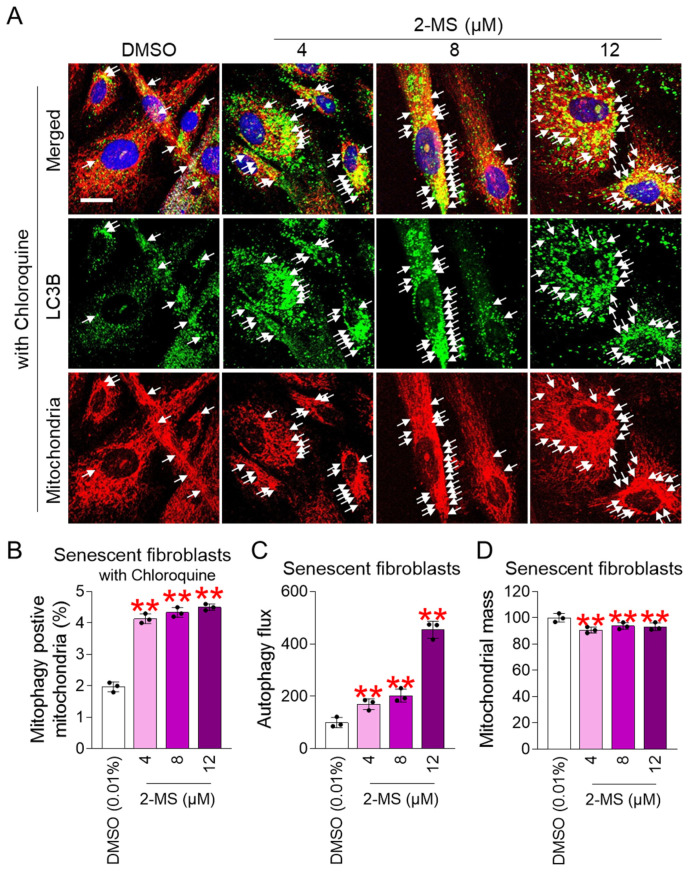
2-MS eliminates dysfunctional mitochondria through mitophagy. (**A**,**B**) Senescent fibroblasts were treated with DMSO (0.01%) or 2-MS (4, 8, 12 μM) at 4-day intervals for 12 days. During drug treatment, senescent fibroblasts were co-treated with chloroquine 24 h before immunostaining. Then, immunostaining for LC3B (green) and mitochondria (red) was performed. The nucleus was stained with Hoechst 33342 (blue). Scale bar 10 μm. Mitophagy is indicated by a white arrow. (**C**,**D**) Autophagy flux or mitochondrial mass was measured in senescent fibroblasts treated with DMSO (0.01%) or 2-MS (4, 8, and 12 μM) for 12 days at 4-day intervals. Statistical analysis was performed using one-way ANOVA followed by Bonferroni’s post hoc test, with results considered significant at ** *p* < 0.01. Data represent the mean ± S.D., *n* = 3.

2-MS treatment (4, 8, and 12 μM) significantly increased co-localization of LC3B and mitochondria compared to the DMSO control (Figure S4; white arrows). To further validate the role of 2-MS in mitophagy, chloroquine (CQ) was used. CQ disrupts lysosomal pH, leading to autophagosome accumulation and increased LC3B–mitochondria colocalization [43]. Consistent with this mechanism, CQ-treated cells exhibited markedly increase in colocalization compared with CQ-untreated cells (Figure 3A; CQ-treated cells, Figure S3; CQ-untreated cells). Notably, the group co-treated with CQ and 2-MS (4, 8, and 12 μM) showed a significant increase in co-localization compared to the group co-treated with CQ and DMSO (Figure 3A,B; white arrows). These results suggest that 2-MS activates mitophagy.

Next, autophagic flux was measured to assess mitophagy activation by 2-MS. Autophagic flux, defined as the rate at which autophagy clears damaged organelles such as mitochondria [44], was significantly increased in senescent fibroblasts treated with 2-MS (4, 8, and 12 μM) compared with the DMSO control (Figure 3B). These results provide quantitative evidence that 2-MS promotes the activation of mitophagy.

Because we observed that 2-MS-mediated increase in autophagy flux, we measured mitochondrial mass to assess whether damaged mitochondria were also removed by increased autophagy. In senescent fibroblasts treated with 2-MS (4, 8, and 12 μM), mitochondrial mass was significantly reduced compared to the DMSO control (Figure 3C).

To determine whether 2-MS affects mitochondrial dynamics, we examined the expression of key proteins involved in mitochondrial fission and fusion. Compared to the DMSO control, 2-MS treatment (4, 8, and 12 μM) did not alter the expression level of dynamin-related protein 1 (Drp1), a fission-inducing GTPase [45] (Figure S4). Furthermore, 2-MS did not affect the expression of essential fusion proteins, including the outer membrane GTPase mitofusin 1 and the inner membrane GTPase optic atrophy 1 (OPA1) (both long and short forms, L-OPA1 and S-OPA1) [45] (Figure S4). Collectively, these results suggest that 2-MS does not regulate mitochondrial fission or fusion.

### 3.4. 2-MS Improves Senescence-Associated Phenotypes

**Figure 4:**
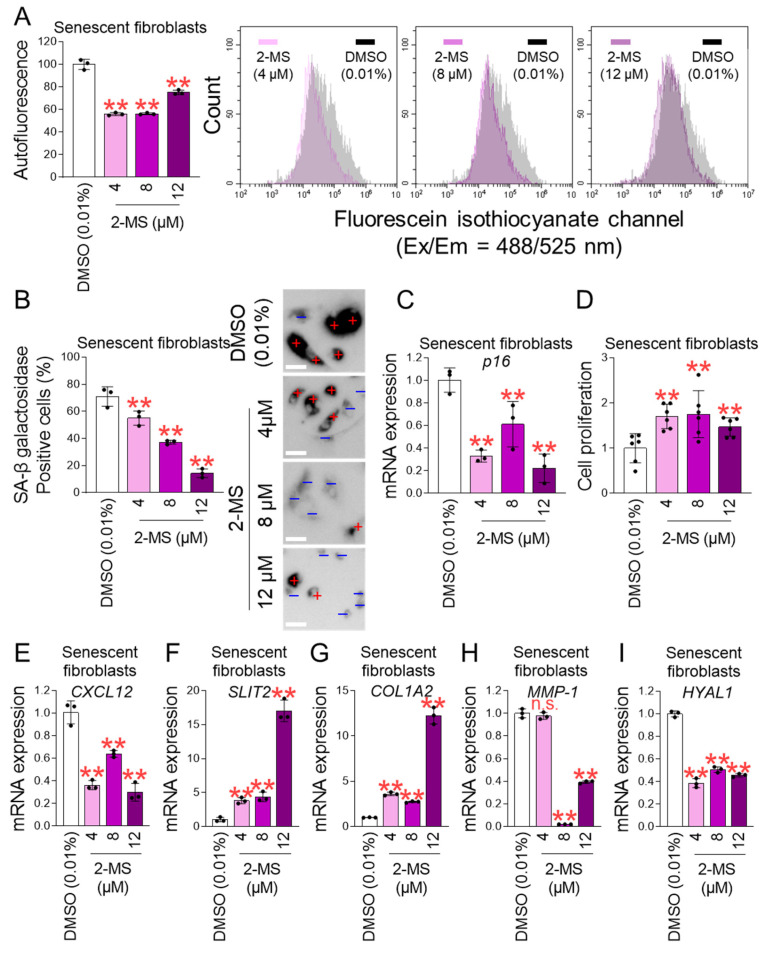
2-MS improves senescence-associated phenotypes. Senescent fibroblasts treated with DMSO (0.01%) or 2-MS (4, 8, 12 μM) at 4-day intervals for 12 days. Then, autofluorescence (**A**), senescence-associated β-galactosidase (SA-β-gal) (**B**), *p16* (**C**), cellular proliferation (**D**), *CXCL12* (**E**), *SLIT2* (**F**), *COL1A2* (**G**), *MMP-1* (**H**), and *HYAL1* expression (**I**) were measured. (**A**) Representative flow graphs of autofluorescence. (**B**) + indicates SA-β galactosidase-positive cells. − indicates SA-β galactosidase negative cells. Scale bar: 10 μm. Statistical analysis was performed using one-way ANOVA followed by Bonferroni’s post hoc test, with results considered not significant (n.s.) or significant at ** *p* < 0.01. Data represent the mean ± S.D., *n* = 3.

Restoration of mitochondrial activity is essential for rejuvenating senescent cells [46,47,48,49,50]. During senescence, lipofuscin (a cross-linked protein residue formed by iron-catalyzed oxidation) accumulates in lysosomes [51]. To determine whether 2-MS rejuvenates senescence, we examined its effect on lipofuscin levels. Intracellular accumulation of lipofuscin was assessed by autofluorescence measurement [52]. Senescent fibroblasts treated with 2-MS (4, 8, and 12 μM) exhibited significantly reduced autofluorescence compared with DMSO-treated controls, indicating that 2-MS effectively suppresses lipofuscin accumulation (Figure 4A). However, the effect of 2-MS on autofluorescence was not dose-dependent. Specifically, treatment with 2-MS at 12 μM concentration did not produce a greater reduction in autofluorescence compared to the lower concentrations (4 and 8 μM). These findings suggest that the effective concentration range of 2-MS for reducing autofluorescence is confined to a narrow window.

Next, we evaluated the effect of 2-MS on SA-β-gal activity, a hallmark of cellular senescence [53]. Treatment with 2-MS significantly reduced the proportion of SA-β-gal–positive cells in a dose-dependent manner (4, 8, and 12 μM) compared with the DMSO control (Figure 4B).

Cellular senescence is defined by irreversible cell cycle arrest, triggered by ROS-induced DNA damage and protein oxidation [54]. Since p16 inhibits CDK4/6 activity and prevents cell cycle progression from G1 to S phase [55], we assessed *p16* expression following 2-MS treatment. Senescent fibroblasts treated with 2-MS (4, 8, and 12 μM) exhibited significantly reduced *p16* expression compared with DMSO-treated controls, indicating that 2-MS alleviates cell cycle arrest (Figure 4C). However, the effect of 2-MS on *p16* expression did not exhibit a linear dose-dependence. Instead, it demonstrated a biphasic, bell-shaped response curve, wherein the intermediate concentration (8 μM) was less effective than both the lower (4 μM) and higher (12 μM) doses. This non-linear dynamic suggests that 2-MS may engage distinct molecular targets to modulate *p16* expression [56].

The finding that 2-MS significantly reduces *p16* expression led us to investigate the effects of 2-MS on cell proliferation. Compared with the DMSO control, treatment with 2-MS (4, 8, and 12 μM) significantly increased cell proliferation, confirming the 2-MS-induced decrease in *p16* expression (Figure 4D).

The senescence-associated secretory phenotype (SASP) is characterized by the secretion of cytokines and chemokines by senescent cells [57]. Within the mitochondrial matrix, ROS interact with mitochondrial superoxide dismutase to generate hydrogen peroxide [58]. This hydrogen peroxide can diffuse across the mitochondrial outer membrane, where it damages cytoplasmic proteins and promotes the release of SASP factors, including the pro-inflammatory chemokine CXCL12 (CXC motif chemokine 12) [59]. Treatment of senescent fibroblasts with 2-MS (4, 8, and 12 μM) significantly reduced *CXCL12* expression compared to DMSO-treated controls, indicating that 2-MS downregulates the inflammatory SASP (Figure 4E). However, the effect of 2-MS on *CXCL12* expression did not exhibit a linear dose-dependence. Instead, it demonstrated a biphasic, bell-shaped response curve, wherein the intermediate concentration (8 μM) was less effective than both the lower (4 μM) and higher (12 μM) doses. This non-linear dynamic also suggests that 2-MS may engage distinct molecular targets to modulate *CXCL12* expression [56].

Following the observation that 2-MS downregulates inflammatory SASP factors, we investigated its effects on anti-inflammatory mediators. Slit Guidance Ligand 2 (SLIT2), which exerts anti-inflammatory effects through the signaling of nuclear factor kappa-light-chain-enhancer of activated B cells [60], was examined after 2-MS treatment. Senescent fibroblasts treated with 2-MS (4, 8, and 12 μM) exhibited significantly increased *SLIT2* expression compared with DMSO-treated controls, indicating that 2-MS upregulates anti-inflammatory factors (Figure 4F).

Decreased collagen synthesis compromises the structural support of the skin barrier and is a hallmark of skin aging [61]. To determine whether 2-MS inhibits this process, we examined the expression level of *type I collagen alpha 2* (*COL1A2*). Compared to the DMSO control, 2-MS treatment (4, 8, and 12 μM) significantly increased *COL1A2* expression (Figure 4G). These results suggest that 2-MS promotes collagen production and improves the structural integrity of the skin.

Matrix metalloproteinase 1 (MMP-1) impairs skin barrier function by promoting collagen degradation [62]. After confirming that 2-MS promotes collagen synthesis, we investigated its effect on *MMP-1* expression. While 2-MS at a concentration of 4 μM did not alter *MMP-1* expression compared to the DMSO control, concentrations of 8 μM and 12 μM significantly decreased expression (Figure 4H). This suggests that at high concentrations, 2-MS further protects the skin barrier by inhibiting collagen degradation.

Hyaluronidase 1 (HYAL1) degrades hyaluronic acid, a component of the skin, leading to impaired skin barrier function [63]. To determine the effect of 2-MS on *HYAL1* expression, we examined *HYAL1* expression levels. Compared to the DMSO control, 2-MS treatment (4, 8, and 12 μM) significantly decreased *HYAL1* expression (Figure 4I).

Having confirmed that 2-MS improves senescence-associated phenotypes in senescent fibroblasts, we next assessed whether it modulates the same parameters in young fibroblasts. Compared to DMSO-treated young fibroblasts, 2-MS treatment (4, 8, and 12 μM) significantly reduced autofluorescence and mitochondrial ROS levels, consistent with the effects observed in senescent fibroblasts (Figure S6A,B). However, compared to DMSO-treated young fibroblasts, 2-MS treatment (4, 8, and 12 μM) did not alter mitochondrial mass, a result that differs from the effect observed in senescent fibroblasts (Figure S6C). Furthermore, the effect of 2-MS on the senescence marker *p16* was also distinct from that observed in senescent fibroblasts. While basal *p16* expression was unchanged at concentrations of 4 and 12 μM, a significant upregulation was induced at the intermediate concentration of 8 μM (Figure S6D). Finally, 2-MS consistently suppressed *CXCL12* expression and upregulated *SLIT2* expression at all tested concentrations (4–12 μM), directly consistent with the effects observed in senescent fibroblasts (Figure S6E,F).

### 3.5. 2-MS Suppresses ROS-Driven Melanogenesis and Inflammatory Responses

**Figure 5:**
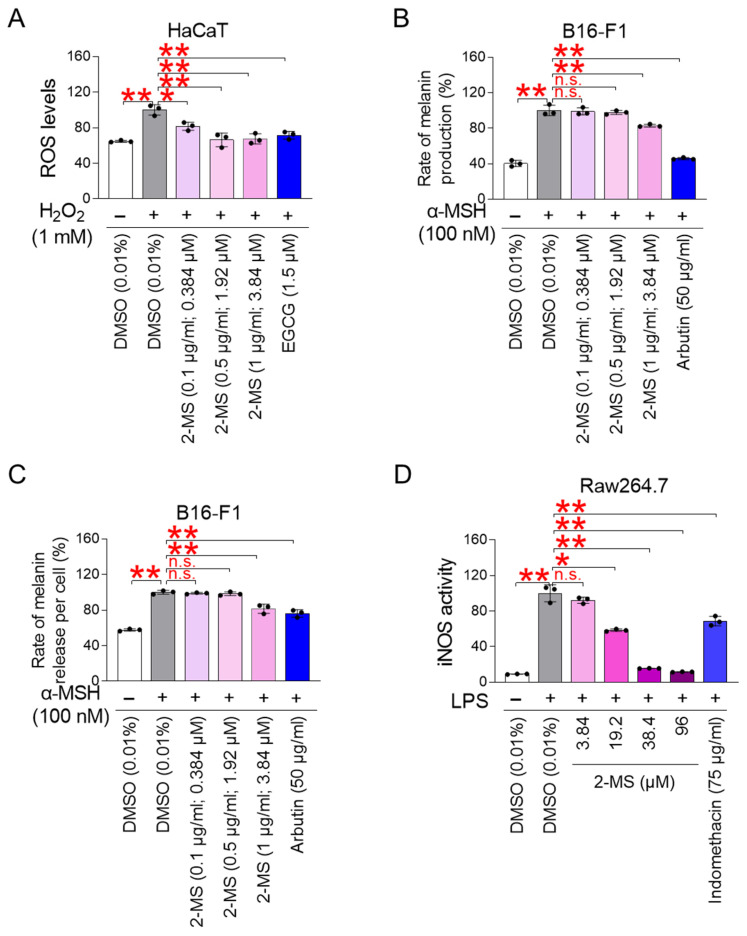
2-MS suppresses ROS-driven melanogenesis and inflammatory responses. (**A**) HaCaT keratinocytes were treated with DMSO (0.01%), 2-MS (0.1, 0.5, and 1 μg/mL; 0.384, 1.92, and 3.84 μM), or epigallocatechin gallate (EGCG; 1.5 μM) for 1 day. Then, HaCaT keratinocytes were exposed with hydrogen peroxide (H_2_O_2_) for 30 min. ROS levels were measured using 20 μM 2′,7′-dichlorodihydrofluorescein diacetate. Statistical analysis was performed using one-way ANOVA followed by Bonferroni’s post hoc test, with results considered significant at * *p* < 0.05 and ** *p* < 0.01. Data represent the mean ± S.D., *n* = 3. (**B**,**C**) B16-F1 cells were stimulated with α-melanocyte-stimulating hormone (α-MSH) for 3 days. Then, B16-F1 cells treated with DMSO (0.01%), 2-MS (0.1, 0.5, and 1 μg/mL; 0.384, 1.92, and 3.84 μM), or arbutin (50 μg/mL) for 1 day. The rate of melanin production and release was measured. Statistical analysis was performed using one-way ANOVA followed by Bonferroni’s post hoc test, with results considered not significant (n.s.) or significant at ** *p* < 0.01. Data represent the mean ± S.D., *n* = 3. (**D**) RAW264.7 macrophages were stimulated with lipopolysaccharide (LPS). Then, RAW264.7 macrophages were treated with DMSO (0.01%), 2-MS (3.84, 19.2, 38.4, and 96 μM), or indomethacin (75 μg/mL) for 1 day. The iNOS activity was measured using Griess assay kit. Statistical analysis was performed using one-way ANOVA followed by Bonferroni’s post hoc test, with results not significant (n.s.) or significant at * *p* < 0.05 and ** *p* < 0.01. Data represent the mean ± S.D., *n* = 3.

Building on the senescence-ameliorating effects of 2-MS in fibroblasts, we next investigated whether these benefits extend to skin cells. Oxidative stress was artificially induced in HaCaT keratinocytes, a widely used skin cell model, using hydrogen peroxide (H_2_O_2_) [64]. H_2_O_2_ treatment significantly increased intracellular ROS levels, suggesting successful induction of oxidative stress (Figure 5A). Epigallocatechin gallate (EGCG), a well-known antioxidant, served as a positive control and effectively suppressed H_2_O_2_-induced ROS accumulation (Figure 5A). Notably, 2-MS treatment (0.1, 0.5, and 1 μg/mL; 0.384, 1.92, and 3.84 μM) significantly reduced ROS levels in H_2_O_2_-treated HaCaT cells, demonstrating its antioxidant activity in skin cells (Figure 5A).

Melanin, the pigment responsible for skin color, is synthesized in specialized melanocytes [65]. Excessive ROS can contribute to hyperpigmentation by overactivating tyrosinase, a key enzyme in melanin production [66]. To examine the effect of 2-MS on melanogenesis, B16-F1 cells, a mouse melanoma-derived melanocyte model, were used. Melanogenesis was induced by treatment with α-melanocyte-stimulating hormone (α-MSH) [67,68], which significantly increased melanin production (Figure 5B). Arbutin, a known melanogenesis inhibitor, served as a positive control [69] and significantly suppressed α-MSH–induced melanin production (Figure 5B). In α-MSH–stimulated B16-F1 cells, treatment with 2-MS significantly reduced melanin production only at the highest tested concentration of 1 μg/mL (3.84 μM), whereas lower doses (0.1 and 0.5 μg/mL; 0.384 and 1.92 μM) had no effect (Figure 5B). These findings indicate that a specific concentration threshold of 2-MS is required to effectively inhibit melanogenesis.

The rate of melanosome transfer from melanocytes to keratinocytes, referred to as the rate of melanin release, is proportional to melanin production [70]. Thus, measuring melanin release provides an indirect assessment of melanin synthesis. To induce melanogenesis, B16-F1 cells were treated with α-MSH [67,68], which significantly increased melanin release (Figure 5C). Arbutin, used as a positive control, significantly suppressed α-MSH–induced melanin release (Figure 5C). In α-MSH–stimulated B16-F1 cells, treatment with 2-MS significantly reduced melanin release only at the highest tested concentration of 1 μg/mL (3.84 μM), whereas lower doses (0.1 and 0.5 μg/mL; 0.384 and 1.92 μM) had no effect (Figure 5C). These findings indicate that a specific concentration threshold of 2-MS is required to effectively inhibit the release of melanin.

Skin inflammation is a major contributor to skin aging, as it damages tissue through sustained inflammatory signaling [10]. Nitric oxide (NO) amplifies inflammation by promoting the production of pro-inflammatory cytokines [71]. The inducible nitric oxide synthase (iNOS), the enzyme responsible for NO synthesis, is primarily expressed in immune cells such as macrophages [72]. To evaluate the anti-inflammatory effects of 2-MS, RAW264.7 macrophages were stimulated with lipopolysaccharide (LPS) to induce iNOS production [73]. LPS treatment significantly increased iNOS activity (Figure 5D). Indomethacin, a nonsteroidal anti-inflammatory drug, served as the positive control and effectively reduced LPS-induced iNOS activity (Figure 5D). Similarly, 2-MS treatment (1, 5, 10 and 25 μg/mL; 3.84, 1.92, 38.4, and 96 μM) decreased LPS-stimulated iNOS activity in a dose-dependent manner, demonstrating its anti-inflammatory potential (Figure 5D).

## 4. Discussion

ROS are highly reactive molecules that damage cellular organelles. During OXPHOS, electrons can leak from complexes I and III, partially reducing oxygen to form superoxide radicals, a major source of intracellular ROS [74]. As cells undergo senescence, electron leakage from these complexes increases, further elevating ROS production and damaging the electron transport chain and other organelles, creating a vicious cycle that accelerates senescence [75,76]. Therefore, reducing mitochondrial ROS is a key strategy for mitigating cellular senescence. In this study, we demonstrated that 2-MS, an active component of *P. cuspidatum* extract, effectively decreases mitochondrial ROS production. Mechanistically, 2-MS enhances electron transport efficiency, as evidenced by increased OXPHOS efficiency, which in turn restores mitochondrial function and reduces reliance on glycolysis. To our knowledge, this is the first study to show that 2-MS reduces mitochondrial ROS, highlighting its potential as a cornerstone for anti-senescence strategies targeting mitochondrial oxidative stress.

Natural compounds, such as green tea extract and aloe vera, have been shown to mitigate skin aging by acting as antioxidants and promoting collagen production [77]. These compounds are frequently incorporated into cosmetics because they are generally less likely to cause adverse skin effects compared with synthetic ingredients [78]. However, identifying the active components is critical, as natural compounds often consist of complex mixtures. Selecting the active ingredients can minimize the inclusion of potentially toxic or unnecessary compounds in skin-aging formulations [77]. In this study, we identified 2-MS as the primary antioxidant component of *P. cuspidatum* extract. 2-MS is a naturally occurring anthraquinone derivative within the naphthoquinone family [20,21]. Anthraquinone derivatives contain hydroxyl and methoxy groups, which can donate electrons or hydrogen atoms to neutralize ROS [79]. Consequently, the reduction in mitochondrial ROS generation observed in this study may be attributed to the antioxidant properties of 2-MS. This effect was further supported by the ability of 2-MS to reduce artificially induced oxidative stress in skin cell models. Importantly, we demonstrated that 2-MS exerts its antioxidant activity not only through efficient electron transfer in mitochondria but also due to its structural features that enhance ROS scavenging.

Mitophagy is essential for maintaining cellular homeostasis by selectively removing damaged mitochondria [80]. During progressive senescence, mitophagy efficiency declines, leading to the accumulation of dysfunctional mitochondria and further acceleration of senescence [81]. In this study, we found that 2-MS restored mitophagy activity, as evidenced by increased colocalization of mitochondria and autophagosomes after 2-MS treatment. Additionally, 2-MS enhanced autophagic flux, facilitating the removal of damaged organelles, including defective mitochondria, providing quantitative evidence for mitophagy activation. Activation of mitophagy reduced the number of dysfunctional mitochondria, thereby lowering mitochondrial ROS levels. To our knowledge, these findings are the first to demonstrate that 2-MS selectively eliminates defective mitochondria in senescent fibroblasts, revealing a novel mechanism for 2-MS-mediated mitochondrial ROS generation.

While 2-MS effectively promotes mitochondrial autophagy, it does not significantly affect core mitochondrial dynamics, as evidenced by the maintenance of unchanged expression of key mitochondrial fission (Drp1) and fusion (OPA1, mitofusin 1) proteins. Therefore, 2-MS appears to induce mitochondrial quality control through targeted removal rather than restructuring of the mitochondrial network. However, because proper quality control relies on a balance between removal and biogenesis, it is also possible that 2-MS stimulates mitochondrial biogenesis through the mitochondrial biogenesis factor peroxisome proliferator-activated receptor gamma coactivator 1-alpha [82]. Further studies investigating the effects of 2-MS on these biogenesis pathways are needed to fully elucidate the mechanisms of mitochondrial quality control.

ROS-mediated oxidative stress is a major driver of skin inflammation, often associated with elevated expression of senescence-associated secretory phenotype (SASP) factors [83]. These factors disrupt the local microenvironment, promoting chronic inflammation that accelerates cellular senescence [84]. Therefore, interventions that attenuate SASP-induced inflammation are of significant therapeutic interest [85]. In this study, 2-MS significantly reduced the expression of *CXCL12*, a key pro-inflammatory SASP component, while increasing *SLIT2*, an anti-inflammatory factor. These beneficial effects were further supported by the reduction in LPS-stimulated inflammation in skin cell models. Collectively, these findings highlight the therapeutic potential of 2-MS for mitigating inflammatory conditions.

Hyperpigmentation is a prominent visual hallmark of skin aging and is largely driven by ROS-mediated overactivation of tyrosinase, the key enzyme in melanin production [86]. This pathway increases melanogenesis, leading to excess melanin synthesis by melanocytes. Previous studies have indicated that antioxidant compounds can mitigate ROS, potentially reducing melanogenesis [87]. In this study, 2-MS treatment effectively reduced both the rate of melanin production and melanin release, indicating a decrease in overall melanin synthesis. These findings suggest that 2-MS has potential as a cosmetic ingredient for skin aging, although further studies are needed to confirm its efficacy in vivo.

## 5. Conclusions

In summary, we demonstrated that 2-MS effectively reduces mitochondrial ROS in senescent fibroblasts. This ROS-lowering effect is mediated by enhanced electron transport in the ETC, leading to restoration of senescence-associated phenotypes. Additionally, 2-MS ameliorates senescence by mitigating ROS-driven melanogenesis and inflammatory responses. These findings reveal a novel mechanism by which 2-MS restores senescence through mitochondrial ROS reduction. These results provide a foundation for therapeutic or cosmetic interventions aimed at mitigating cellular senescence.

## Figures and Tables

**Table 1:** List of primers.

Target	Orientation	Sequence (5′–3′)	Size (bp)
*36B4* (Accession number: NM_053275)	Forward	CAGCAAGTGGGAAGGTGTAATCC	23
	Reverse	CCCATTCTATCATCAACGGGTACAA	25
*p16* (Accession number: NM_000077.5)	Forward	CTCGTGCTGATGCTACTGAGGA	22
	Reverse	GGTCGGCGCAGTTGGGCTCC	20
*CXCL12* (Accession number: NM_199168.4)	Forward	TCAGCCTGAGCTACAGATGC	20
	Reverse	CTTTAGCTTCGGGTCAATGC	20
*SLIT2* (Accession number: NM_053275)	Forward	CAGAGCTTCAGCAACATGACCC	22
	Reverse	GAAAGCACCTTCAGGCACAACAG	23
*COL1A2* (Accession number: NM_000089.4)	Forward	CCTGGTGCTAAAGGAGAAAGAGG	23
	Reverse	ATCACCACGACTTCCAGCAGGA	22
*MMP-1* (Accession number: NM_002421.4)	Forward	ATGAAGCAGCCCAGATGTGGAG	22
	Reverse	TGGTCCACATCTGCTCTTGGCA	22
*HYAL1*	Forward	GACACGACAAACCACTTTCTGCC	23
(Accession number: NM_007312)	Reverse	ATTTTCCCAGCTCACCCAGAGC	22

**Table 2:** Western blot conditions.

Analysis	Antibody	Catalogue Number	Dilution in PBS	Staining Condition
Drp1 staining	anti-Drp1	A2586; Abclonal	1:200	overnight at 4 °C
	Horseradish peroxidase–conjugated antibody	sc–2357; Santa Cruz biotechnology; Dallas, TX, USA	1:1000	60 min at room temperature
OPA1 staining	anti-OPA1	A9833; Abclonal	1:200	overnight at 4 °C
	Horseradish peroxidase–conjugated antibody	sc–2357; Santa Cruz biotechnology; Dallas, TX, USA	1:1000	60 min at room temperature
Mitofusin 1 staining	anti-mitofusin 1	A12771; Abclonal	1:200	overnight at 4 °C
	Horseradish peroxidase–conjugated antibody	sc–2357; Santa Cruz biotechnology; Dallas, TX, USA	1:1000	60 min at room temperature

## Data Availability

The original contributions presented in the study are included in the article, further inquiries can be directed to the corresponding authors.

## Supplementary Materials

The following supporting information can be downloaded at https://www.mdpi.com/article/10.3390/antiox15030357/s1: Figure S1. Cellular senescence was assessed by quantifying the percentage of senescence-associated β-Galactosidase (SA-β-Gal)-positive cells (A) and the expression levels of the antiproliferative markers *p21* (B) and *p16* (C). Figure S2. Non-mitochondrial respiration, maximal respiration, and ATP-coupled respiration were measured in senescent fibroblasts treated with DMSO (0.01%) or 2-MS (4, 8, and 12 μM) for 12 days at 4-day intervals. Figure S3. The effect of 2-MS on mitochondrial function in young fibroblasts. Figure S4. Immunofluorescence analysis of LC3B (green fluorescence) and mitochondria (red fluorescence) in senescent fibroblasts treated with DMSO (0.01%) or 2-MS (4, 8, 12 μM) at 4-day intervals for 12 days. Figure S5. 2-MS does not regulate the expression of proteins involved in mitochondrial fission or fusion. Figure S6. The effect of 2-MS on senescence-associated phenotypes in young fibroblasts.
