# Supplementary material for: 2-Methoxystypandrone from Polygonum cuspidatum Rejuvenates Senescence by Reducing Mitochondrial ROS
Source: Antioxidants (Basel). 2026 Mar 11;15(3):357. doi: 10.3390/antiox15030357 (PMC13023934; doi:10.3390/antiox15030357)
Supplement: Supplementary file 1 [file antioxidants-15-00357-s001.zip › antioxidants-4139089-supplementary.pdf]

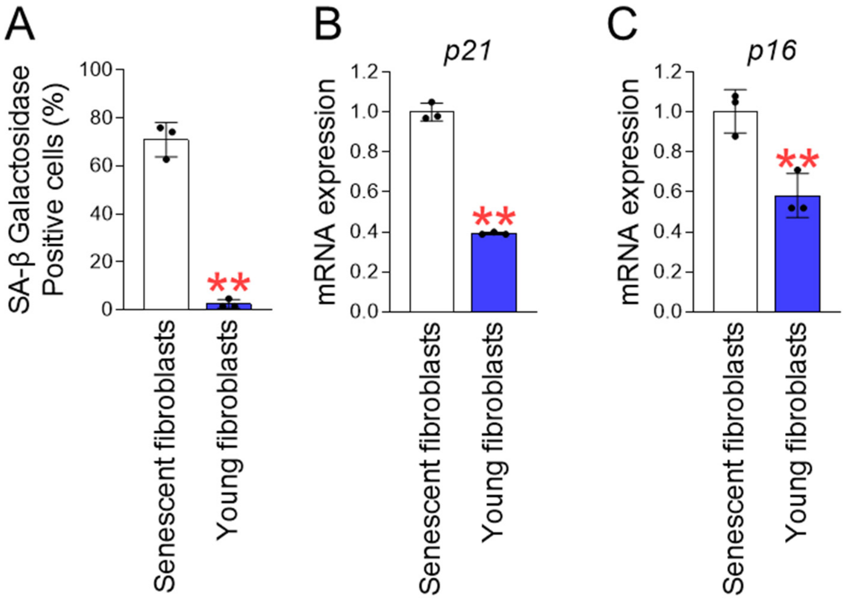

Figure S1. Cellular senescence was assessed by quantifying the percentage of senescence-associated β-Galactosidase (SA-β-Gal)-positive cells (A) and the expression levels of the antiproliferative markers *p21* (B) and *p16* (C). Statistical analysis was performed using Student's *t*-test, with results considered significant at ** $p < 0.01$. Data represent the mean ± S.D., $n = 3$.

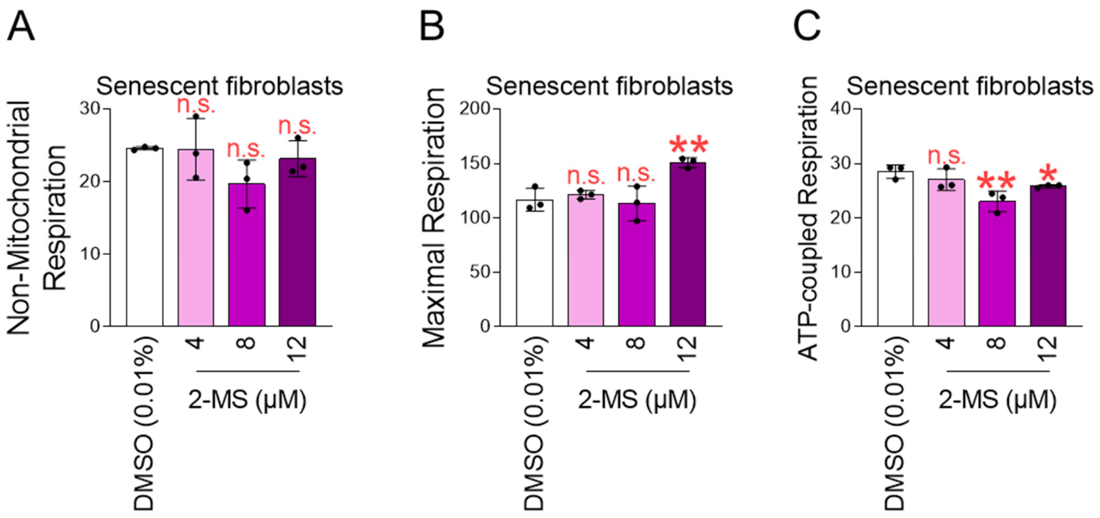

Figure S2. Non-mitochondrial respiration, maximal respiration, and ATP-coupled respiration were measured in senescent fibroblasts treated with DMSO (0.01%) or 2-MS (4, 8, and 12 μM) for 12 days at 4-day intervals. Statistical significance of non-mitochondrial respiration, maximal respiration, and ATP-coupled respiration was assessed using one–way ANOVA followed by Bonferroni's post–hoc test, with results considered not significant (n.s.) or significant at $*p < 0.05$ and $**p < 0.01$. Data represent the mean ± S.D., $n = 3$.

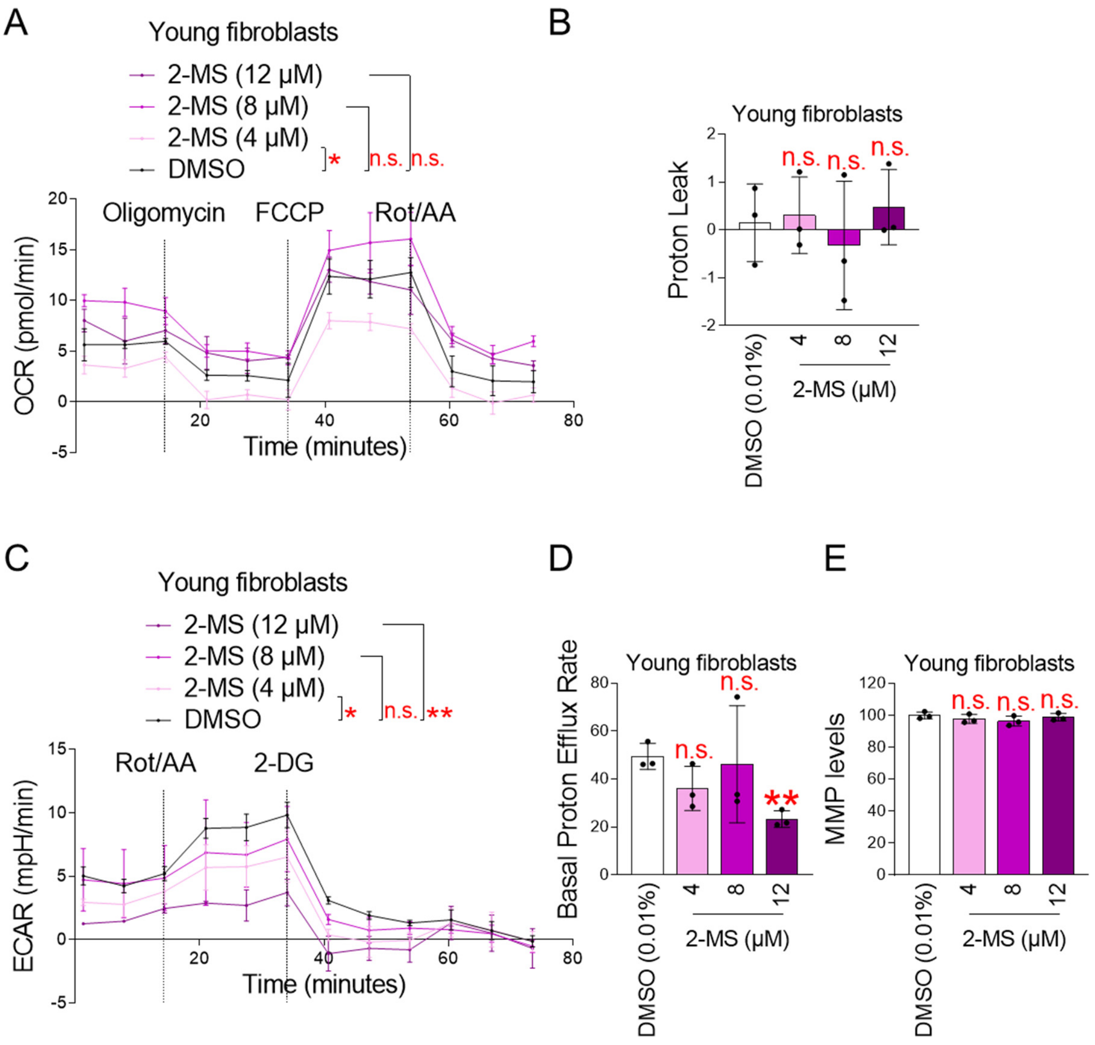

Figure S3. The effect of 2-MS on mitochondrial function in young fibroblasts. Oxygen consumption rate (OCR; pmol/min) and proton leak were measured using the Seahorse XF Mito Stress Test Kit (**A**,**B**). ECAR and basal proton efflux rate were measured using the Seahorse XF Glycolytic Rate Assay Kit (**C**,**D**). Flow cytometric analysis of mitochondrial membrane potential (MMP) using JC-1 (**E**). Statistical significance of OCR and ECAR was assessed using two–way ANOVA followed by Bonferroni's post hoc test, with results considered not significant (n.s.) or significant at * $p < 0.05$ and ** $p < 0.01$. Data represent the mean ± S.D., $n = 5$. Statistical significance of proton leak, basal proton efflux, and MMP levels was assessed using one–way ANOVA followed by Bonferroni's post–hoc test, with results considered not significant (n.s.) or significant at **$p < 0.01$. Data represent the mean ± S.D., $n = 3$.

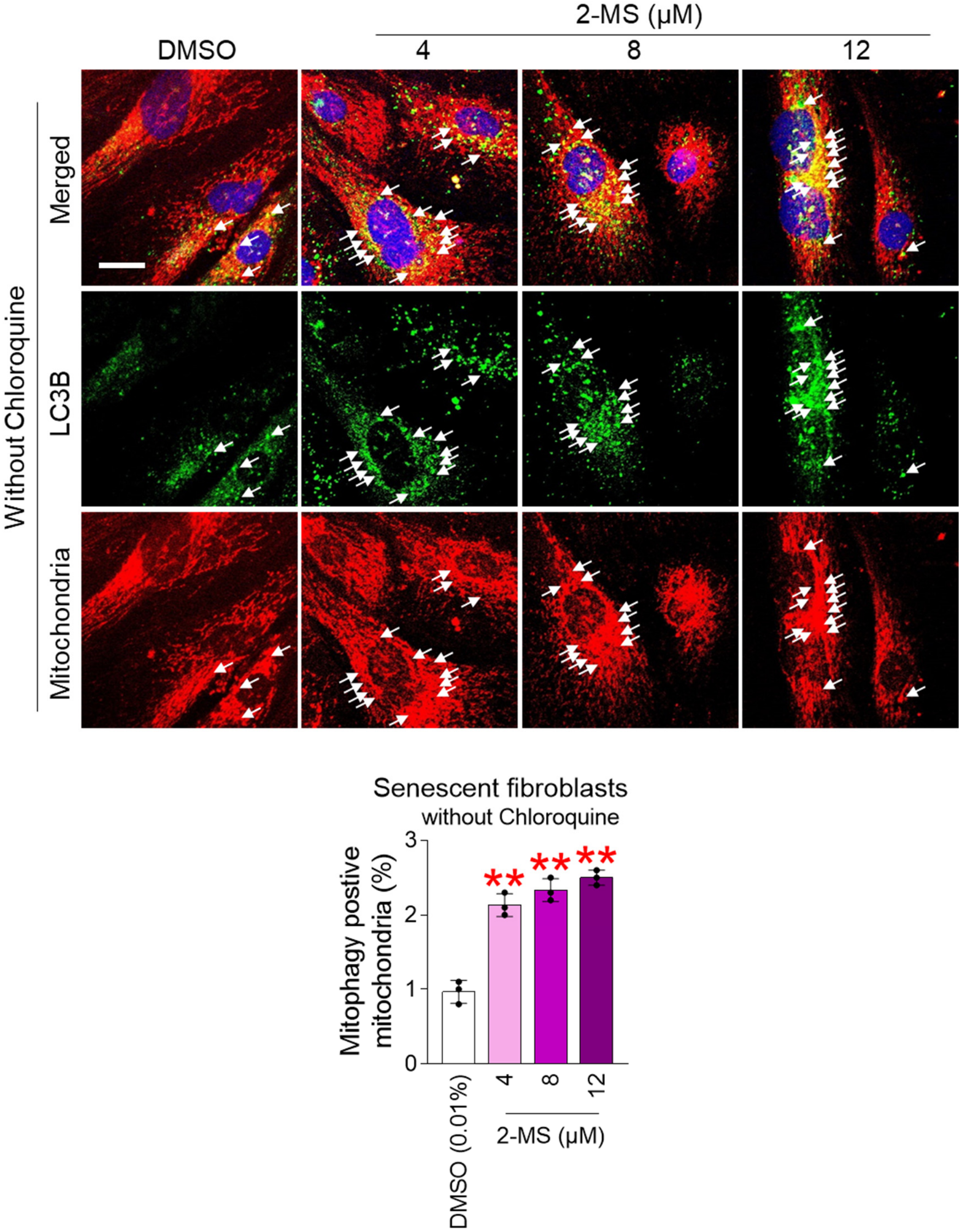

Figure S4. Immunofluorescence analysis of LC3B (green fluorescence) and mitochondria (red fluorescence) in senescent fibroblasts treated with DMSO (0.01%) or 2-MS (4, 8, 12 μM) at 4-day intervals for 12 days. During treatment, senescent fibroblasts were not co-treated with 20 μM chloroquine 24 h before Immunostaining. Scale bar 10 μm. Mitophagy is indicated by a white arrow. Statistical significance of mitophagy positive mitochondria was assessed using one–way ANOVA followed by Bonferroni's post–hoc test, with results considered significant at $^{**}p < 0.01$. Data represent the mean ± S.D., $n = 3$.

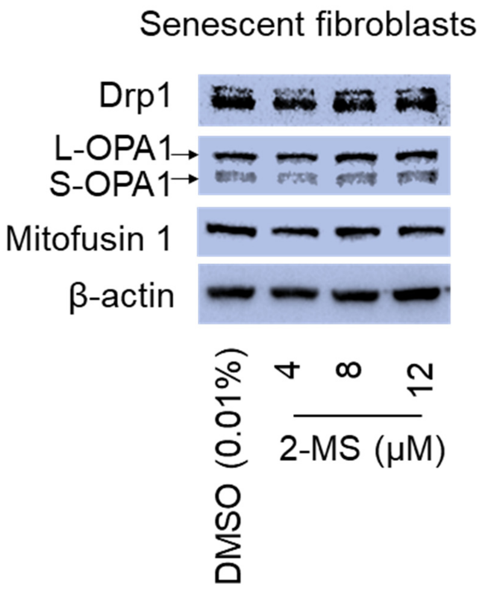

Figure S5. 2-MS does not regulate the expression of proteins involved in mitochondrial fission or fusion. Compared to the DMSO control, 2-MS treatment (4, 8, and 12 μM) did not alter the expression levels of dynamin-related protein 1 (Drp1), a fission-inducing GTPase. Furthermore, 2-MS did not affect the expression of essential fusion proteins, including the outer membrane GTPase mitofusin 1 and the inner membrane GTPase optic atrophy 1 (OPA1) (both long and short forms, L-OPA1 and S-OPA1).

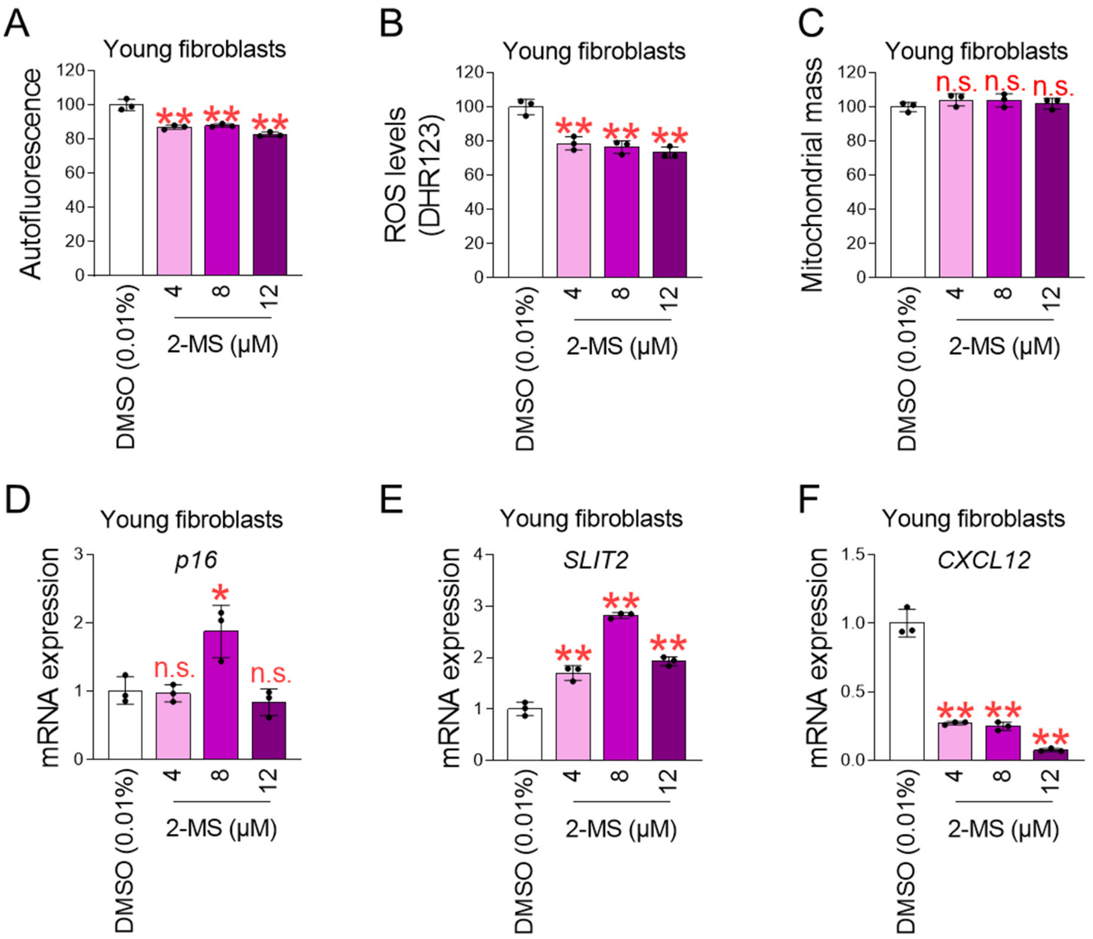

Figure S6. The effect of 2-MS on senescence-associated phenotypes in young fibroblasts. Autofluorescence (**A**), Mitochondrial ROS (**B**), mitochondrial mass (**C**), *p16* (**D**), *CXCL12* (**E**), and *SLIT2* expression (**F**) were measured in young fibroblasts treated with DMSO (0.01%) or 2-MS (4, 8, 12 μM). Statistical analysis was performed using one–way ANOVA followed by Bonferroni's post–hoc test, with results considered not significant (n.s.) or significant at $^*P < 0.05$ and ** $p < 0.01$. Data represent the mean ± S.D., n = 3.
